# EphA2- and HDAC-Targeted Combination Therapy in Endometrial Cancer

**DOI:** 10.3390/ijms25021278

**Published:** 2024-01-20

**Authors:** Robiya Joseph, Santosh K. Dasari, Sujanitha Umamaheswaran, Lingegowda S. Mangala, Emine Bayraktar, Cristian Rodriguez-Aguayo, Yutuan Wu, Nghi Nguyen, Reid T. Powell, Mary Sobieski, Yuan Liu, Mark Seungwook Kim, Sara Corvigno, Katherine Foster, Pahul Hanjra, Thanh Chung Vu, Mamur A. Chowdhury, Paola Amero, Clifford Stephan, Gabriel Lopez-Berestein, Shannon N. Westin, Anil K. Sood

**Affiliations:** 1 Department of Gynecologic Oncology and Reproductive Medicine, The University of Texas MD Anderson Cancer Center, Houston, TX 77030, USA; rjoseph7@mdanderson.org (R.J.); santy2407@gmail.com (S.K.D.); sumamaheswaran@mdanderson.org (S.U.); lsmangala@mdanderson.org (L.S.M.); ebayraktar@mdanderson.org (E.B.); wyutuandr@gmail.com (Y.W.); yliu32@mdanderson.org (Y.L.); mkim3@mdanderson.org (M.S.K.); scorvigno@mdanderson.org (S.C.); fosterki13@gmail.com (K.F.); phanjra@mdanderson.org (P.H.); tvu9@mdanderson.org (T.C.V.); mamur@utexas.edu (M.A.C.); swestin@mdanderson.org (S.N.W.); 2 UTHealth Houston Graduate School of Biomedical Sciences, The University of Texas MD Anderson Cancer Center, Houston, TX 77030, USA; 3 Department of Experimental Therapeutics, The University of Texas MD Anderson Cancer Center, Houston, TX 77030, USA; crodriguez2@mdanderson.org (C.R.-A.); pamero@mdanderson.org (P.A.); glopez@mdanderson.org (G.L.-B.); 4 High-Throughput Research and Screening Center, Center for Translational Cancer Research, Texas A&M Health Science Center, Institute of Biosciences and Technology, Houston, TX 77030, USA; nnguyen@tamu.edu (N.N.); repowell@tamu.edu (R.T.P.); msobieski@tamu.edu (M.S.); cstephan@ibt.tamhsc.edu (C.S.)

**Keywords:** endometrial cancer, EphA2, histone deacetylase

## Abstract

Endometrial cancer is the most frequent malignant tumor of the female reproductive tract but lacks effective therapy. EphA2, a receptor tyrosine kinase, is overexpressed by various cancers including endometrial cancer and is associated with poor clinical outcomes. In preclinical models, EphA2-targeted drugs had modest efficacy. To discover potential synergistic partners for EphA2-targeted drugs, we performed a high-throughput drug screen and identified panobinostat, a histone deacetylase inhibitor, as a candidate. We hypothesized that combination therapy with an EphA2 inhibitor and panobinostat leads to synergistic cell death. Indeed, we found that the combination enhanced DNA damage, increased apoptosis, and decreased clonogenic survival in Ishikawa and Hec1A endometrial cancer cells and significantly reduced tumor burden in mouse models of endometrial carcinoma. Upon RNA sequencing, the combination was associated with downregulation of cell survival pathways, including senescence, cyclins, and cell cycle regulators. The Axl-PI3K-Akt-mTOR pathway was also decreased by combination therapy. Together, our results highlight EphA2 and histone deacetylase as promising therapeutic targets for endometrial cancer.

## 1. Introduction

Endometrial cancer is the most common gynecologic malignancy, with increasing rates of mortality [1]. The survival outcomes are poor in patients with advanced disease, and hence, there is an urgent need for safe and effective treatment options to improve the survival of patients with advanced-stage endometrial cancer [2]. The receptor tyrosine kinase ephrin type-A receptor 2 (EphA2) is highly expressed in many types of human cancer [3,4,5] but found at very low levels in most normal epithelial tissues, indicating its potential application in cancer therapy. Mounting evidence has demonstrated the role of EphA2 in tumor growth and metastasis [6]; further, EphA2 overexpression is associated with poor prognosis in several cancers, including endometrial cancer [7]. In preclinical models of ovarian, breast, and pancreatic cancer, the inhibition of EphA2 decreased tumor growth and increased survival [7]. Our previous studies demonstrated excellent delivery of EphA2 siRNA through 1,2-dioleoyl-sn-glycero-3-phosphatidylcholine (DOPC) neutral nanoliposomes (EPHARNA) to mouse ovarian tumors, which resulted in decreased tumor burden [8]. Additionally, the combination of EPHARNA with paclitaxel significantly reduced tumor growth compared to treatment with paclitaxel alone.

Previously, we demonstrated a synergistic interaction between EphA2- and Wee1-targeted therapies in endometrial cancer models through high-throughput chemical screens, which served as a platform for this study. We previously confirmed the synergistic effects of the combination in both in vitro and in vivo studies [9]. Since synergistic combinations with EphA2-targeted therapy remain limited, we sought to identify additional therapeutic combinations. Here, we identified a synergistic partner to EphA2-targeted therapy in endometrial cancer, namely, the HDAC (histone deacetylase) inhibitor panobinostat (LBH589). HDACs are enzymes that catalyze the removal of acetyl functional groups from both histone and nonhistone proteins [10]. The aberrant expression of HDACs has been linked to a variety of malignancies, including solid and hematological tumors, and a high level of HDACs is associated with advanced disease and poor outcomes in patients [11].

We hypothesized that combined EphA2 and HDAC inhibition leads to synergistic endometrial cancer cell death. To test our hypothesis, we examined the antitumor effects of an EphA2 inhibitor and panobinostat, as individual agents and in combination, in mouse models of endometrial cancer. We observed synergistic interaction between the EphA2 inhibitor and panobinostat and established the underlying mechanisms responsible for the synergy. Our results justify further development of this combination strategy to treat endometrial carcinoma.

## 2. Results

### 2.1. Identification of Rational Combinations with EphA2 Inhibition Using High-Throughput Drug Screen

**Figure 1:**
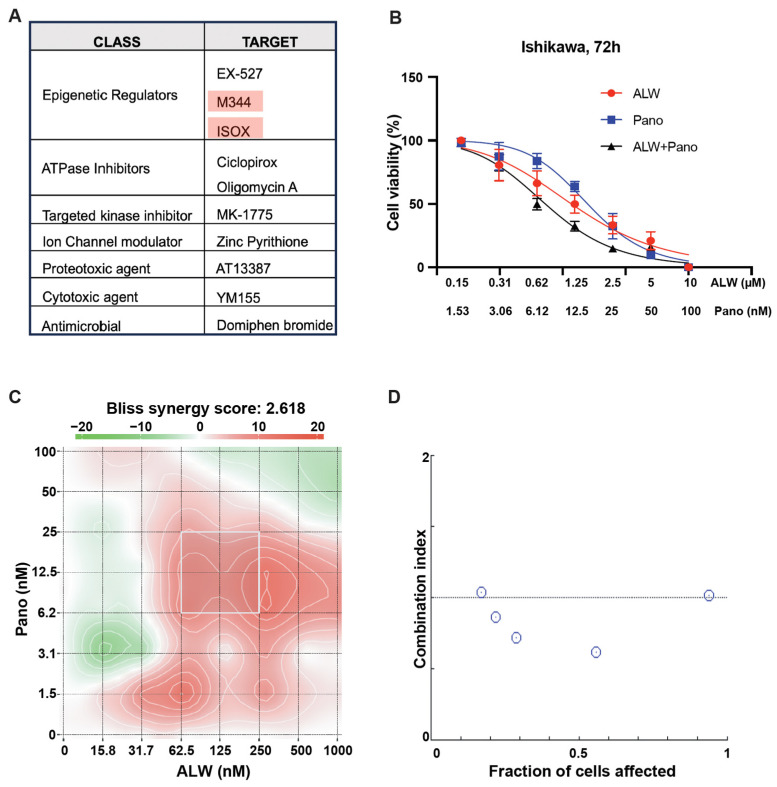
Identification of rational combinations to EphA2 inhibition. (**A**) Identification of synergistic partners for EphA2 using a high throughput screen wherein the top 10 hits from the chemical screen are shown. (**B**) Effect of EphA2 inhibitor ALW and HDAC inhibitor panobinostat (Pano) on the viability of Ishikawa cells, assessed at 72 h. (**C**) Graphical representation of 2D synergy maps showing the MTT cell viability assay results from the SynergyFinder Bliss independence model combinatorial analysis, wherein red regions represent synergy and green regions represent antagonism. (**D**) Plots showing fraction of cells affected and combination index values for ALW and panobinostat showing synergy (the white circles represent combination index values less than 1).

We hypothesized that candidate small-molecule drugs would overcome resistance to EphA2 inhibition, enhancing its therapeutic benefit. Using the Ishikawa endometrial cancer cell line transfected with either control siRNA or EphA2 siRNA, we performed a high-throughput drug screen using two drug libraries (the Broad Collection-Informer Set and Selleck Bioactives Collection) containing a total of 1510 drugs comprising FDA-approved agents, investigational agents, and bioactive molecules. Among the top 10 hits from the screen, epigenetic regulators (EX-527, M344, and ISOX) were the class most commonly represented, followed by ATPase inhibitors (ciclopirox and oligomycin A) and targeted kinase inhibitor (MK-1775) (Figure 1A). Among the three epigenetic regulators identified as candidates, both M344 and ISOX are HDAC inhibitors. Since panobinostat is an FDA-approved HDAC inhibitor, it was chosen for further study as a potential synergistic partner for EphA2 inhibition. The EphA2 inhibitor ALW-II-41-27 (hereafter ALW) was also selected for further study due to its antitumor activity in many solid tumors [12,13].

For in vitro studies, we chose two endometrial cancer cell lines with high expression of EphA2 (Ishikawa and Hec1A). After these cancer cells were treated with the individual drugs or the combination for 72 h, their viability was assessed using MTT assays. Upon treatment with the combination, a significant and dose-dependent decrease in cell viability was seen in both Ishikawa (Figure 1B) and Hec1A cells (Supplemental Figure S1A). We also observed that cell viability was significantly lower following combination therapy than individual drug treatments at every dose tested. Panobinostat demonstrated the greatest synergistic interaction score when combined with ALW in Ishikawa cells (Bliss synergy score of 2.618, most synergistic area score of 8.11) (Figure 1C) and in Hec1A cells (Bliss synergy score of 6.101, most synergistic area score of 19.6) (Supplemental Figure S1B). To further test the drug-drug interaction, we used the median effect equation to derive combination index values using SynergyFinder (version 3.0) and CompuSyn software (version 1.0.1) and observed that the drug combination produced a synergistic effect (Figure 1D) in Ishikawa cells. This observation was also validated using Hec1A cells (Supplemental Figure S1C).

### 2.2. EphA2- and HDAC-Targeted Combination Therapy Results in Enhanced DNA Damage, Increased Apoptosis, and Decreased Clonogenic Survival in Endometrial Cancer Cells

**Figure 2:**
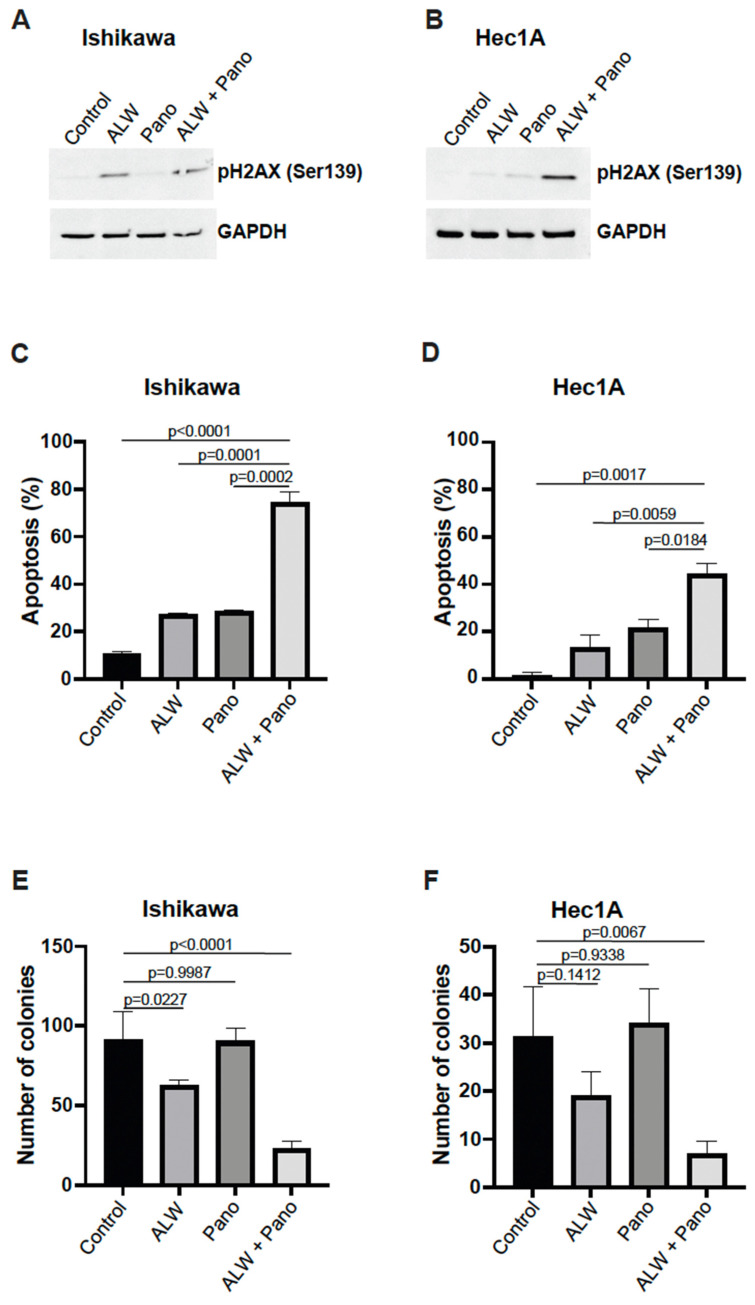
Combination therapy decreased cell survival by facilitating apoptotic cell death. (**A**,**B**) Western blot analysis for γH2AX phosphorylation in Ishikawa (**A**) and Hec1A (**B**) cells that were untreated or treated with ALW, panobinostat (Pano), or the combination. (**C**,**D**) Flow cytometry analysis of apoptosis in Ishikawa (**C**) and Hec1A (**D**) cells that were untreated or treated with ALW, panobinostat, or both after 48 h. (**E**,**F**) Clonogenic colony formation assay in Ishikawa (**E**) and Hec1A (**F**) cells that were untreated or treated with ALW, panobinostat, or both. Comparisons were performed using one-way analysis of variance (ANOVA) with the Tukey post hoc test for multiple comparisons (for more than two groups).

To further validate the effect of the combination treatment on cell survival of endometrial cancer cells, we assessed H2AX phosphorylation, a sensitive marker for double-strand DNA breaks, which can lead to apoptotic cell death. We observed increased H2AX phosphorylation at S139 after combination treatment compared to individual treatment, suggesting the presence of excessive double-strand DNA breaks during cell death, in Ishikawa (Figure 2A) and Hec1A (Figure 2B) cells.

Next, we further evaluated the effects of the combination treatment using an annexin V/propidium iodide (PI)-based apoptosis assay with flow cytometry and observed a significantly higher percentage of apoptosis with the combination treatment compared with individual drug treatments in both Ishikawa (Figure 2C) and Hec1A (Figure 2D) cells. Additionally, we assessed early and late apoptotic patterns by analyzing annexin V-positive PI-negative fraction (early apoptosis) and annexin V-positive and PI-positive fraction (late apoptosis) and observed significantly more frequent early and late apoptotic events with the combination treatment compared to individual drugs in Ishikawa cells (Supplemental Figure S2A) and Hec1A cells (Supplemental Figure S2B). We then analyzed p21 expression, a known cell cycle regulator [14], and observed higher p21 expression in the Pano and in the combination groups when compared to control and ALW alone in Hec1A cells (48 h) (Supplemental Figure S2C).

Furthermore, to determine the effect of combination therapy on clonogenic survival, we performed colony formation assays using both cell lines. We observed that the combination treatment resulted in significantly lower colony-forming ability compared to no treatment and the individual treatments in both Ishikawa (Figure 2E) and Hec1A (Figure 2F) cells.

### 2.3. EPHARNA and Panobinostat Reduce Endometrial Cancer Growth in Orthotopic Models

**Figure 3:**
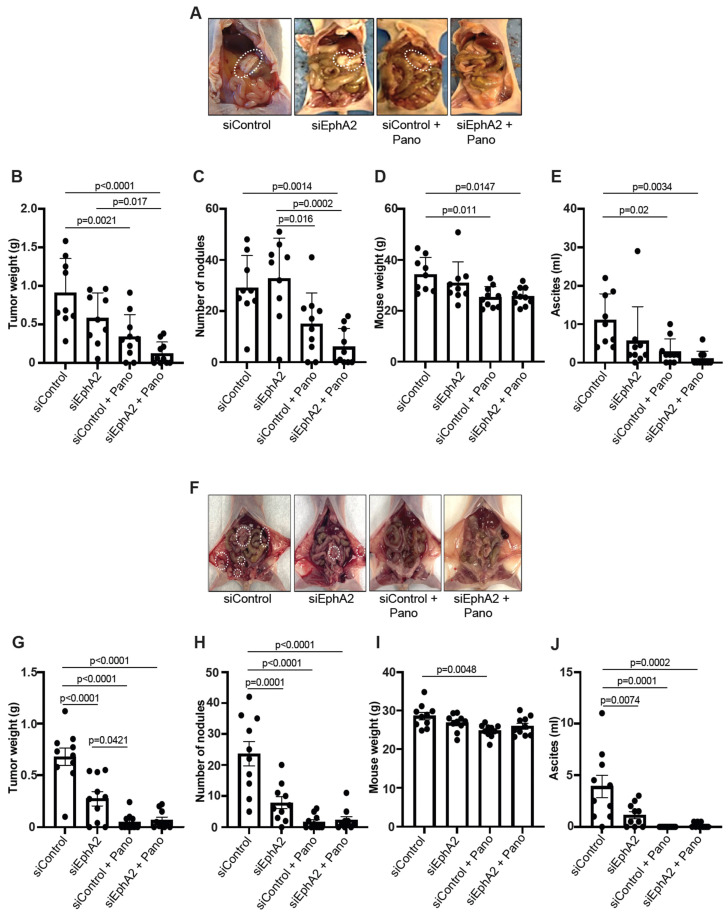
Assessment of antitumor effects of EphA2- and HDAC-targeted therapy in orthotopic endometrial cancer xenograft models. Representative images of tumor burden in mice with Ishikawa-Luc (**A**) and Hec1A-Luc cells (**F**) tumors with siControl-DOPC nanoparticles (NPs) and siEphA2-DOPC NPs and/or panobinostat therapy (the white dashed circles indicate tumor nodules). Tumor weights (**B**), number of nodules (**C**), mouse body weight at the end of the experiment (**D**), and ascites volume (**E**) after therapy in Ishikawa-Luc mouse model. Tumor weights (**G**), number of nodules (**H**), mouse body weight (**I**), and ascites volume (**J**) in Hec1A-Luc mouse model. Comparisons were performed using one-way ANOVA with the Tukey post hoc test for multiple comparisons (for more than two groups).

We used orthotopic endometrial cancer models with luciferase-expressing Ishikawa (Ishikawa-Luc) and Hec1A (Hec1A-Luc) cells to study the antitumor effects of siEphA2-DOPC nanoliposomes (EPHARNA) and panobinostat in vivo. In the Ishikawa-Luc model, tumor nodules were established mainly in the uterus, with a few metastases to the peritoneal wall, and mesenteric, and gastric areas (Figure 3A). Mice treated with the EPHARNA and panobinostat combination had significantly lower tumor weight (Figure 3B) with fewer tumor nodules (Figure 3C) and decreased mouse body weight (Figure 3D) due to significantly lower volume of ascites (Figure 3E) compared with control siRNA and individual treatment groups. EPHARNA monotherapy did not reach significance in any measure compared to the control group, whereas the panobinostat monotherapy did result in significantly lower tumor volume, body weight, and ascites volume compared to the control group.

In the Hec1A-Luc model, where the tumors were again localized in the uterus with some metastases to peritoneal, mesenteric, and gastric areas (Figure 3F), the individual drugs and the combination therapy resulted in a significant decrease in tumor burden (Figure 3G), tumor nodules (Figure 3H), and ascitic volume (Figure 3J) compared to the control siRNA group. No significant difference was observed in mouse body weight except for the panobinostat monotherapy group compared to the control group (Figure 3I).

### 2.4. EphA2- and HDAC-Targeted Combination Therapy Downregulates Axl-PI3K-Akt-mTOR Pathway Signaling in Endometrial Cancer

**Figure 4:**
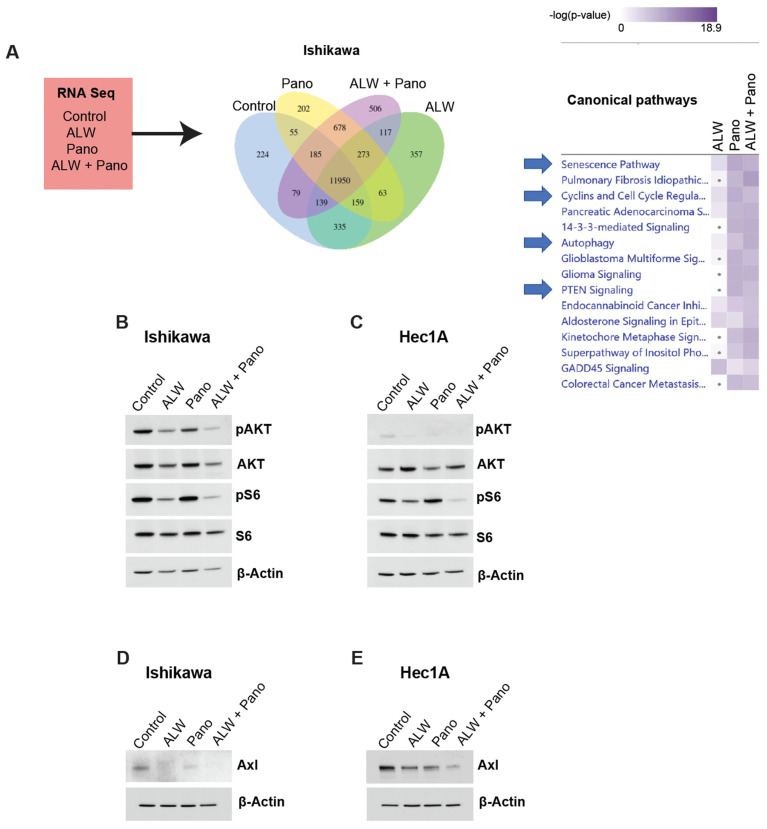
RNA-Seq and IPA for identification of potential downstream molecular mechanisms associated with synergistic interaction of EphA2 and HDAC inhibition. (**A**) RNA-Seq strategy and comparative analysis of pathways downregulated with combination therapy in Ishikawa cells. (**B**,**C**) Validation of RNA-Seq analysis of AKT pathway repression in Ishikawa (**B**) and Hec1A (**C**) cells; additionally phosphorylated S6 levels as an indicator of mTOR downregulation in Ishikawa and Hec1A cells are shown. (**D**,**E**) Validation of Axl as the upstream regulator of PI3K/Akt/mTOR signaling pathway in Ishikawa (**D**) and Hec1A (**E**) cells at 48 h in untreated and treated conditions.

We performed RNA sequencing (RNA-Seq) analysis and subsequent Ingenuity Pathway Analysis (IPA) to identify potential mechanisms associated with the synergistic interaction of EphA2 and HDAC inhibition. To better interpret the observed synergy between the drugs, we performed a comparative analysis to identify enriched canonical pathways that were high under EphA2 inhibition alone and low under EphA2- and HDAC-targeted combination therapy. Canonical pathways (e.g., senescence pathway, cyclins and cell cycle regulators, and PTEN signaling) were downregulated, and other cell survival pathways (e.g., autophagy) were upregulated with the combination (Figure 4A).

AKT is regulated by EphA2 and mediates cell survival [15,16,17,18]. Hence, to better understand the molecular mechanisms associated with the synergism of EphA2 and HDAC inhibition, we examined pAKT levels in Ishikawa cells under untreated, monotherapy, and combination-treated conditions. pAKT levels were lower with the combination therapy compared to the monotherapy and untreated conditions, suggesting decreased survival of cancer cells with combination therapy (Figure 4B). Similar effects were also observed in the Hec1A model (Figure 4C).

Furthermore, we observed decreased phosphorylated S6 levels in both Ishikawa and Hec1A cells, suggesting downregulation of mTOR signaling. Axl, an upstream regulator of the PI3K-AKT-mTOR pathway, was also decreased with the combination treatment in both Ishikawa (Figure 4D) and Hec1A (Figure 4E) cells at 48 h of treatment compared to the untreated and monotherapy conditions. This observation was also noted at 24 h of treatment in Ishikawa cells (Supplemental Figure S3A), but not at 24 h of treatment in Hec1A cells (Supplemental Figure S3B). Thus, the combination of EphA2 and HDAC inhibition in endometrial cancer likely causes DNA damage, increased apoptosis, decreased clonogenic survival, and downregulation of the Axl-PI3K-AKT-mTOR pathway.

## 3. Discussion

In this study, we identified and validated panobinostat as a synergistic partner to EphA2-targeted therapy for endometrial cancer. Synergistic interaction between the EphA2 inhibitor ALW and the HDAC inhibitor panobinostat was observed in vitro to lead to enhanced DNA damage and impaired cancer cell survival. The combination therapy further showed enhanced antitumor efficacy in mouse xenograft models of endometrial cancer.

Upon investigating the associated mechanisms, we discovered cell survival pathways including the senescence pathway, cyclins and cell cycle regulators, and PTEN signaling to be downregulated with the combination therapy. EphA2 is known to regulate the PI3K-AKT signaling pathway in many cancer types [15,16,17,18], which prompted us to examine phosphorylated AKT. We observed decreased phosphorylated AKT expression in cells treated with the combination compared to monotherapy or the control. This decreased expression was accompanied by downregulation of Axl in the combination therapy group, which ultimately disrupted downstream cellular responses responsible for cell growth and survival. This could explain the enhanced DNA damage associated with the combination and associated apoptotic cell death. In various cancers, downregulation of Axl has been shown to inhibit downstream AKT phosphorylation, which inversely affected cell growth and survival, proliferation, migration, and metastasis [19,20]. It was previously reported that PI3K/pAkt/pS6 signaling is a major pathway downstream of Axl [20,21,22]. Additionally, it was also reported that Axl is prone to ubiquitin-mediated proteasomal degradation [23,24]. Therefore, it is possible that the observed decrease in the phosphorylation of AKT and S6 could be due to PI3K/AKT pathway inhibition associated with the ubiquitin-mediated proteasomal degradation of Axl.

EphA2 is considered an important molecular target for clinical translation in cancer treatment [25]. Several compounds targeting EphA2 were evaluated and tested in clinical studies of cancers in which the oncogenic function of EphA2 is well established [26]. Despite extensive data and preclinical validation to support its significance, several challenges persist, and novel measures for improving the effects of EphA2-targeted therapy are necessary. Likewise, HDAC inhibitors increase levels of histone acetylation in cancer cells and are promising anticancer agents, with the capacity to inhibit cancer growth, trigger apoptosis, and reverse cellular differentiation in cancer cells [27]. Nonetheless, additional research is necessary to establish their therapeutic role and clinical utility. Here, we demonstrated the synergy and therapeutic potential of EphA2 and HDAC inhibition in endometrial cancer.

## 4. Materials and Methods

### 4.1. Cell Lines and Culture

Cell lines Hec1A (RRID: CVCL_0293) and Ishikawa (RRID: CVCL_2529) were obtained from American Type Culture Collection (ATCC) and the University of Texas MD Anderson Cancer Center Characterized Cell Line Core, respectively. These cell lines were routinely screened for mycoplasma and were validated by short tandem repeat fingerprinting at the MD Anderson core facility. Hec1A were grown in McCoy’s 5A medium (HyClone, Logan, UT, USA), and Ishikawa were grown in Dulbecco’s modified Eagle’s medium (HyClone), supplemented with 10% fetal bovine serum (Sigma-Aldrich, St. Louis, MO, USA) and 0.1% gentamicin sulfate (Gemini Bioproducts, West Sacramento, CA, USA). Cell culture was carried out in a humidified incubator containing 5% CO_2_ at 37  °C. The experiments were performed with cells at 70% to 80% confluence and cultured for fewer than 20 passages for in vitro work and for fewer than 10 passages for in vivo experiments.

### 4.2. siRNA Transfection

Hec1A and Ishikawa cells were plated in 6-well plates at densities selected to reach 60% to 70% confluence overnight. Each well received a combination of 1.3 µg of siRNA in 150 µL of reduced serum medium (Opti-MEM, Thermo Fisher Scientific, Waltham, MA, USA). In a separate tube, 8 µL of Lipofectamine RNAiMAX transfection reagent (Thermo Fisher Scientific, Waltham, MA, USA) was incubated in 150 µL of Opti-MEM for 5 min. This siRNA/media mixture was added dropwise to the transfection reagent mixture, vortexed, and then incubated for 15 min at room temperature. The cells to be transfected were washed once with 1×PBS, and then 900 µL of Opti-MEM and 300 µL of siRNA mixture were added dropwise to each well. The 6-well plates were gently swirled and placed in the incubator for 4 to 6 h, after which the transfection medium was replaced with complete media. The transfected cells were used for two experiments: high-throughput screening and protein estimation by Western blot analysis. For the screening, after 24 h of transfection, the cells were trypsinized, counted, and seeded in clear-bottom 384-well plates; for the Western blot, cells were collected at 48 and 72 h after transfection.

### 4.3. High-Throughput Chemical Screening

The chemical screens were conducted by the Gulf Coast Consortia’s Combinatorial Drug Discovery Program at the Institute of Biosciences and Technology in Texas A&M Health Science Center, Houston, TX, USA. The transfected Ishikawa cells (siControl or siEphA2) were screened with two drug library collections: the Broad Collection–Informer Set, consisting of 358 compounds, and the Selleck Bioactives Collection, consisting of 1150 compounds (https://ibt.tamu.edu/cores/high-throughput/core-libraries/approved-drugs.html, accessed on 12 July 2019). A total of 800 cells per well was suspended in 50 µL of media and seeded into black 384-well µClear plates (Greiner Bio-One International, Monroe, NC, USA) using a Multidrop Combi liquid dispenser (Thermo Fisher Scientific). The plates were kept at room temperature for 60 min before being moved into a cell culture incubator, and the cells were grown overnight at 37 °C in a humidified chamber with >95% relative humidity and 5% CO_2_. The next day, 50 nL of drugs was transferred into each well using an Echo 550 acoustic dispensing platform (Labcyte, San Jose, CA, USA). As a control, a nontreated plate was immediately fixed with 4% paraformaldehyde, followed by nuclei staining with 4′,6-diamidino-2-phenylindole (DAPI) at the start of drug treatment, designated as day 0. The drug libraries were tested at three concentrations (1 µM, 0.1 µM, and 0.01 µM) with a fixed volume of dimethyl sulfoxide (DMSO) (0.1% *v*/*v*) with two biological replicates. Each assay plate contained the aforementioned fixed concentration of the drugs in addition to two positive controls (etoposide and dasatinib) and a negative control (0.1% DMSO). The plates were then fixed with 0.4% paraformaldehyde, and nuclei were stained with DAPI using an integrated HydroSpeed plate washer (Tecan Life Sciences, Männedorf, Switzerland) and Multidrop Combi dispenser after 72 h. Plates were then imaged on an IN Cell Analyzer 6000 laser-based confocal imaging platform (GE Healthcare Bio-Sciences, Marlborough, MA, USA), and nuclei were counted using IN Cell Developer Toolbox software (version 1.6). The response of cells to the drug screen was evaluated by a curve-fitting calculation and a calculation of area-under-the-curve values.

### 4.4. Cell Viability Assay

Cells were plated in a 96-well plate at a starting density of 2000 cells per well for Hec1A cells and 1000 cells per well for Ishikawa cells in biological triplicates. The cells were used to study the cytotoxicity elicited by the EphA2 inhibitor ALW-II-41-27 (ALW; ApexBio Technology, Houston, TX, USA) and panobinostat (Selleckchem, Houston, TX, USA) both as single agents and in combination. After 24 h of incubation, the medium was aspirated and replaced with 100 µL of fresh medium containing serial dilutions of individual drugs. Following 72 h of incubation, the medium was aspirated, and cells were incubated with 0.05% MTT solution for 1 h. The experiments were performed in triplicate. The supernatant was removed, formazan crystals were dissolved in 100 µL of DMSO, and the plates were read at 570 nm using a uQuant microplate spectrophotometer (BioTek, Winooski, VT, USA). Dose–response curves were plotted using Prism 8.0.0 (GraphPad Software, San Diego, CA, USA), and the combination index was determined by CompuSyn software (ComboSyn, combosyn.com, accessed on 12 July 2019). The synergy assessment was performed using the Bliss model in the SynergyFinder web application (https://synergyfinder.fimm.fi, accessed on 29 July 2019). A score greater than 10 indicates synergy, additivity scores are between −10 and 10, and antagonism scores are less than −10. Ishikawa cells were treated with DMSO, 0.5 µM ALW, 0.25 µM panobinostat, and the combination of 0.5 µM ALW and 0.25 µM panobinostat; Hec1A cells were treated with DMSO, 1 µM ALW, 0.5 µM panobinostat, and the combination of 1 µM ALW and 0.5 µM panobinostat.

### 4.5. Colony Formation

Cells were grown in the incubator for 10 to 14 days with the indicated treatments at a density of 500 to 1000 cells per well in a 12-well plate for both Hec1A and Ishikawa cells. The plates were fixed with a solution containing glutaraldehyde (6.0%, *v*/*v*) and crystal violet (0.5%, *w*/*v*) for 15 to 20 min at room temperature to fix the colonies. The crystal violet fixing solution was decanted, and the plates were washed in water 5 times and left to dry at room temperature. Images of the colonies were acquired using a camera, and the number of colonies was documented.

### 4.6. Apoptosis Studies

The apoptosis assay was performed using an FITC Annexin V Apoptosis Detection Kit I (BD Biosciences, Franklin Lakes, NJ, USA). The control and drug-treated cells were trypsinized, mixed with the cell supernatant (to collect all the cells for this study), spun down and washed with PBS, and later analyzed by flow cytometry to study the extent of apoptosis.

### 4.7. Western Blotting

The cells were trypsinized and spun down at 2000 rpm for 5 min and washed with ice-cold PBS. The cells were then pelleted at 3000 rpm for 3 min and lysed in radioimmunoprecipitation assay (RIPA) buffer supplemented with protease and phosphatase inhibitors. Protein quantification was performed using a Pierce BCA protein assay kit (Thermo Fisher Scientific). A total of 20 µg of protein across all samples was heated at 95 °C for 10 min, subjected to sodium dodecyl sulfate–polyacrylamide gel electrophoresis (8–12%), transferred onto a nitrocellulose membrane, incubated in 5% milk (in Tris-buffered saline–Tween 20 (TBS-T)) for 1 h, and then incubated overnight in the primary antibodies. The blots were washed with TBS-T thrice for 15 min each and then incubated with the secondary antibodies (1:2500 dilution, GE Healthcare, Chicago, IL, USA) for 1 h. The immunoblot images were captured using an Azure Biosystems imaging machine (Azure Biosystems, Dublin, CA, USA) after the addition of enhanced chemiluminescence substrate (Thermo Fisher Scientific) for 1 min. Quantitation of Western blots (Supplemental Figure S4) was performed using ImageJ software (version 1.53, National Institute of Health, Bethesda, MD, USA).

Antibodies: Apoptosis and DNA damage WB Cocktail (pH2A.X/GAPDH/cleaved PARP) (1:250 dilution; Abcam, Cambridge, UK); anti-phospho S6 (1:1000 dilution; Cell Signaling Technology, Danvers, MA, USA), anti-S6 (1:1000 dilution; Cell Signaling Technology), anti-AKT (1:1000 dilution; Cell Signaling Technology); anti-pAKT (1:1000 dilution; Cell Signaling Technology); anti-Axl (1:1000 dilution; Cell Signaling Technology), anti-GAPDH (1:5000; Thermo Fisher Scientific), and anti-beta-actin (1:3000; Sigma-Aldrich).

### 4.8. Liposomal Nanoparticle Preparation

siRNAs were incorporated into DOPC nanoliposomes as described earlier [8,9]. DOPC and siRNA were mixed at a ratio of 1:10 (*w*/*w*) siRNA:DOPC in the presence of excess tertiary butanol. Tween 20 was then added to the siRNA/DOPC mixture at a ratio of 1:19 (Tween-20:siRNA/DOPC). This mixture was vortexed, frozen in an acetone/dry-ice bath, and lyophilized. Prior to in vivo administration in mice, this preparation was hydrated with PBS (magnesium- and calcium-free) to achieve a final concentration of 5 µg of siRNA in 200 µL volume per dose per mouse.

### 4.9. Mouse Model of Endometrial Cancer

The mouse experiments were approved by the Institutional Animal Care and Use Committee of MD Anderson Cancer Center. The experimental mice were housed at the MD Anderson animal facility under strict pathogen-free conditions. One million Ishikawa-Luc and Hec1A-Luc cells in 100 µL of Hank’s Balanced Salt Solution (HyClone) were injected into the peritoneal cavity of 6- to 8-week-old female mice (Taconic Biosciences, Rensselaer, NY, USA) in two separate in vivo experiments. The experimental mice were randomized after 8 days to four groups (10 mice per group): siControl-DOPC NPs, siEphA2-DOPC NPs, siControl-DOPC NPs with panobinostat (ApexBio Technology), and siEphA2-DOPC NPs with panobinostat. The siRNA-DOPC NPs and panobinostat (10 mg/kg in 2.5% DMSO, 5% PEG400, 5% Tween 80 in 0.9% saline) were given to mice twice a week intraperitoneally. The experimental mice were euthanized; mouse weight, tumor weight, number of nodules, and ascites volume were documented at the termination of the experiment.

### 4.10. RNA-Seq

For RNA-Seq, Hec1A cells were plated in triplicate in 6-well plates at a density of 100,000 cells per well and incubated overnight for 24 h. Cells were then treated with DMSO, 1 µM ALW, 10 nM panobinostat, and the combination of 1 µM ALW and 10 nM panobinostat for 8 h, and RNA was extracted using a Direct-zol RNA Miniprep Plus kit (ZYMO Research, Irvine, CA, USA). RNA quality was assessed by RNA integrity number using a Bioanalyzer (Agilent Technologies, Santa Clara, CA). The RNA samples were shipped to Novogene (Sacramento, CA, USA) for RNA-Seq analysis on the Illumina NovaSeq 6000 platform. Downstream analysis was carried out using hisat2, DEseq2, and ClusterProfiler software (version 3.18.1); Ingenuity Pathway Analysis (Qiagen, Hilden, Germany) was used to perform pathway analysis.

### 4.11. Statistical Analysis

One-way ANOVAs with the Tukey post hoc test were used for multiple comparisons between more than two groups (GraphPad Prism, version 10.0.3). *p* values < 0.05 were considered to be statistically significant.

## 5. Conclusions

The combination of EphA2 and HDAC inhibitors triggered DNA damage and inhibited the survival of endometrial cancer cells. Moreover, the combination therapy demonstrated enhanced antitumor efficacy in mouse xenograft models of endometrial cancer. Collectively, our results reveal a novel synergistic interaction between EphA2- and HDAC-targeted therapies in endometrial cancer. These findings open new avenues to identify and validate novel targeted therapeutics in endometrial cancer.

## Data Availability

The datasets generated during the current study are available from the corresponding author on reasonable request.

## Supplementary Materials

The supporting information can be downloaded at: https://www.mdpi.com/article/10.3390/ijms25021278/s1.
