# Supplementary material for: EphA2- and HDAC-Targeted Combination Therapy in Endometrial Cancer
Source: Int J Mol Sci. 2024 Jan 20;25(2):1278. doi: 10.3390/ijms25021278 (PMC10816153; doi:10.3390/ijms25021278)
Supplement: Supplementary file 1 [file ijms-25-01278-s001.zip › ijms-2778903-captions.pdf]

Supplemental Figure S1: Effect of EphA2 inhibitor ALW and HDAC inhibitor panobinostat as individual agents and in combination on Hec1A cells. (A) The effect of ALW and panobinostat on the viability of Hec1A cells at 72 h. (B) Plots of 2D synergy maps showing results of the MTT cell viability assay from the SynergyFinder Bliss independence model combinatorial analysis (red regions represent synergy, and green regions represent antagonism). (C) Plots showing fraction of cells affected and combination index values for ALW and panobinostat showing synergy in Hec1A cells.

Supplemental Figure S2: Validation of effect of combination treatment on cell survival. Effect of combination therapy on early and late apoptosis in Ishikawa (A) and Hec1A (B) cells at 72 h in untreated or treated conditions. p21 levels in Hec1A cells (C) at 48 h in untreated and treated conditions.

Supplemental Figure S3: Confirmation of Axl/PI3K/Akt/mTOR signaling pathway activity with combination treatment. Axl levels in Ishikawa (A) and Hec1A (B) at 24 h in untreated and treated conditions. (C) Schematic representation of the Axl signaling pathway that regulates cell survival.

Supplemental Figure S4: Quantification of western blots used in this study.
