# Supplementary material for: EphA2- and HDAC-Targeted Combination Therapy in Endometrial Cancer
Source: Int J Mol Sci. 2024 Jan 20;25(2):1278. doi: 10.3390/ijms25021278 (PMC10816153; doi:10.3390/ijms25021278)

Supplemental figure S1

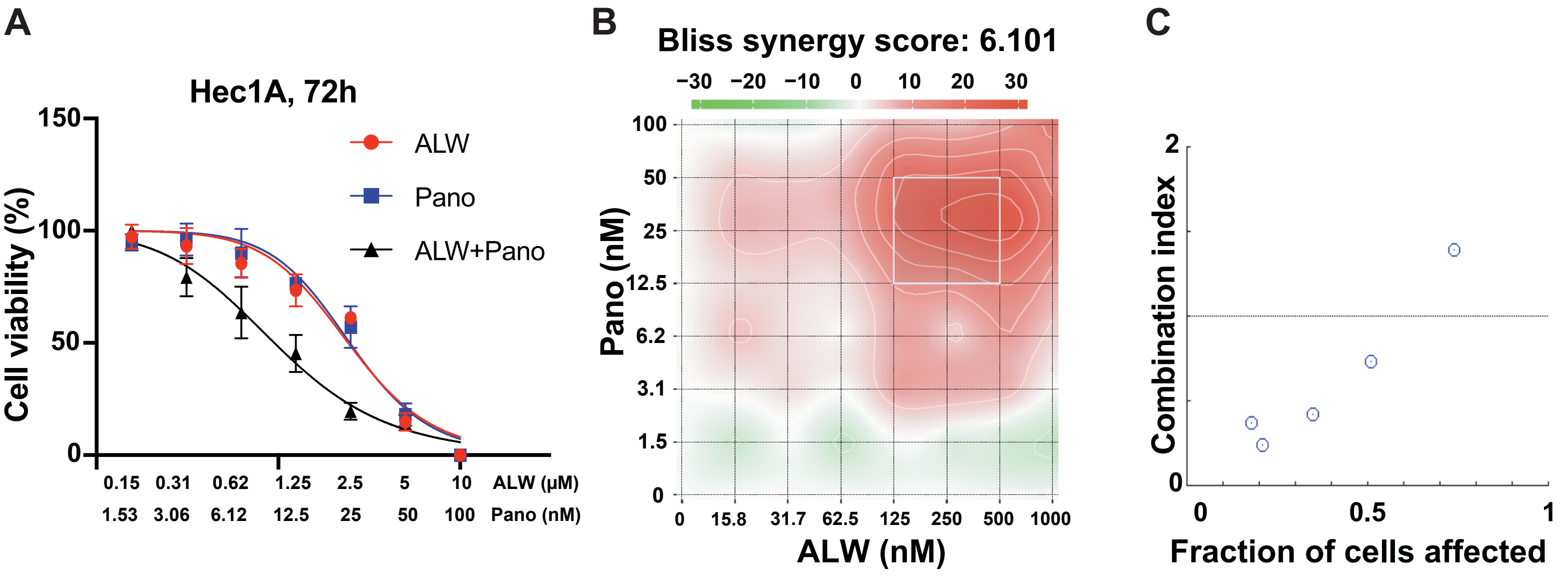

Supplemental figure S2

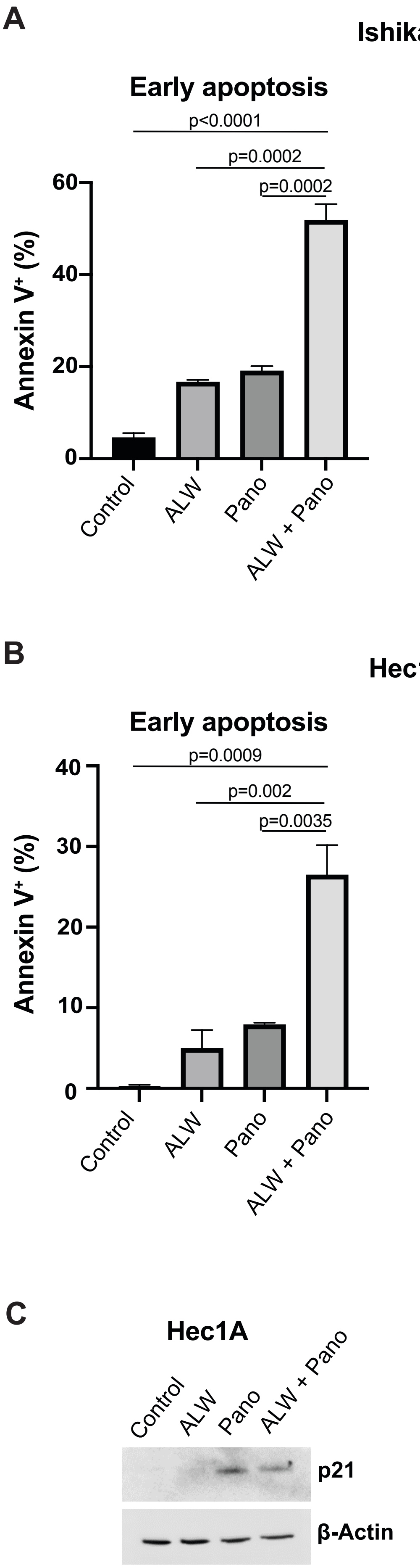

Supplemental figure S3

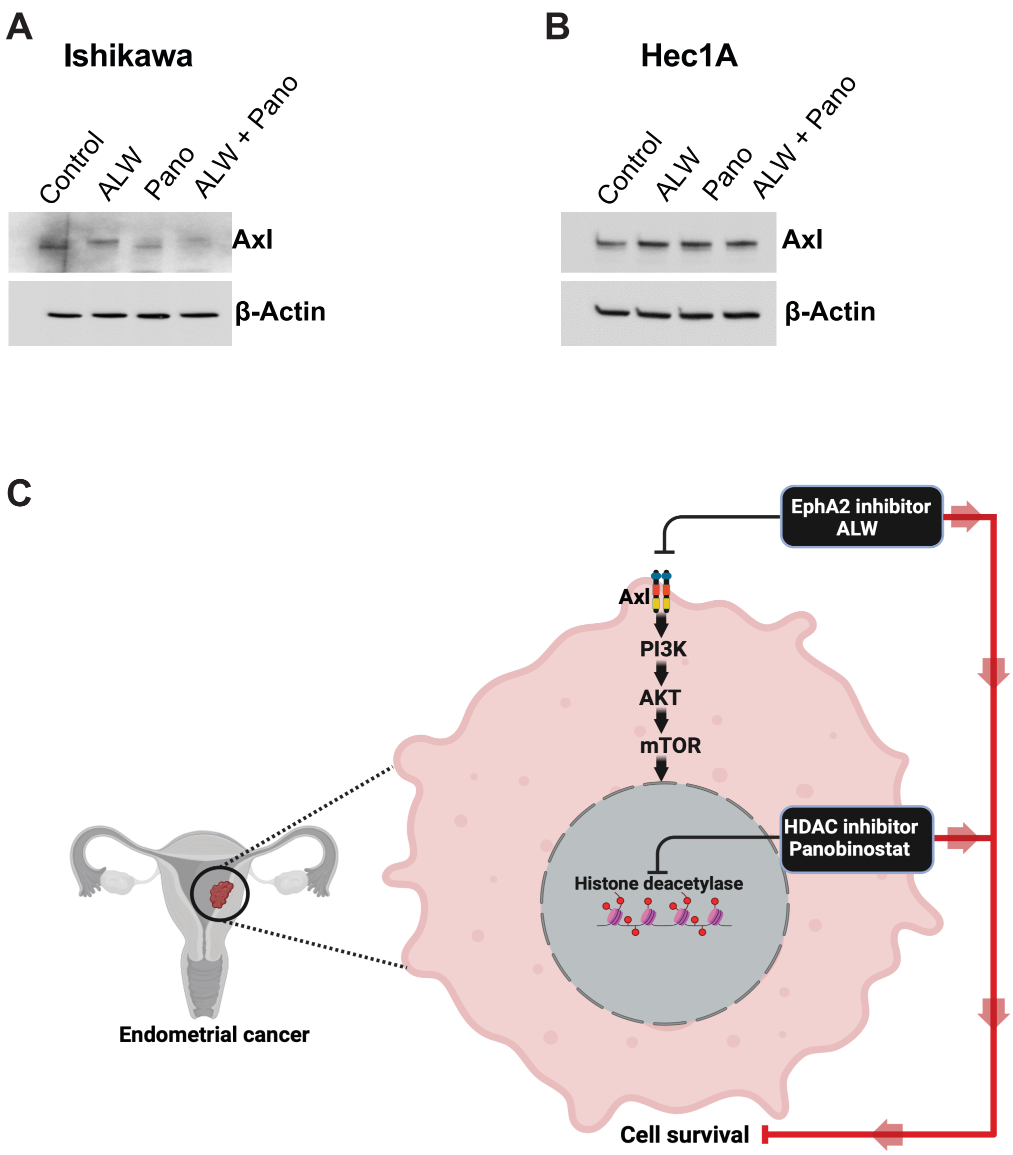

Supplemental figure S4

Relative density associated with Figure 2

A

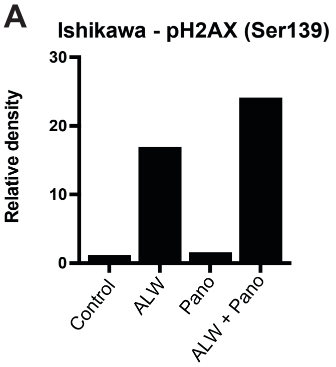

B

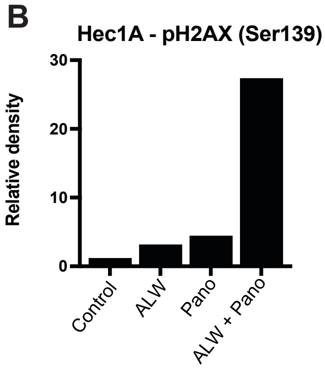

Relative density associated with Figure 4

B

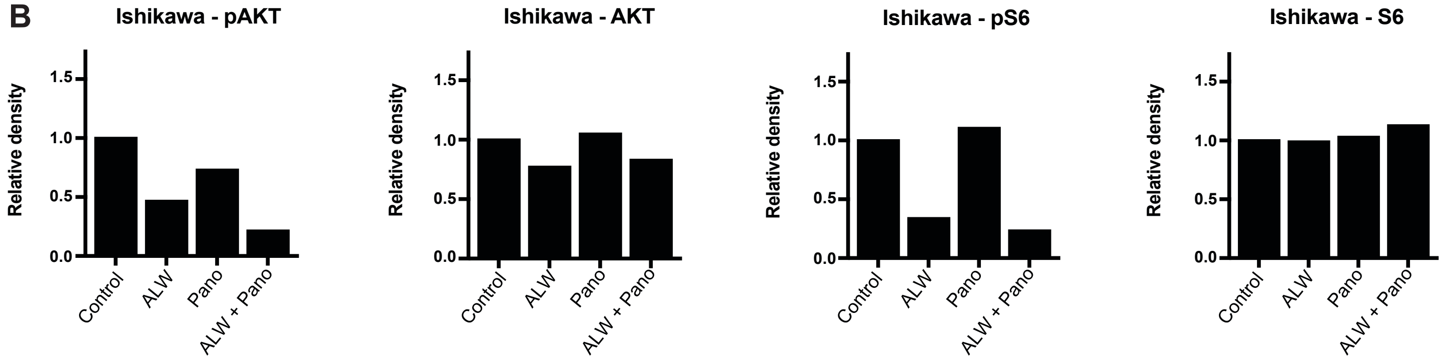

C

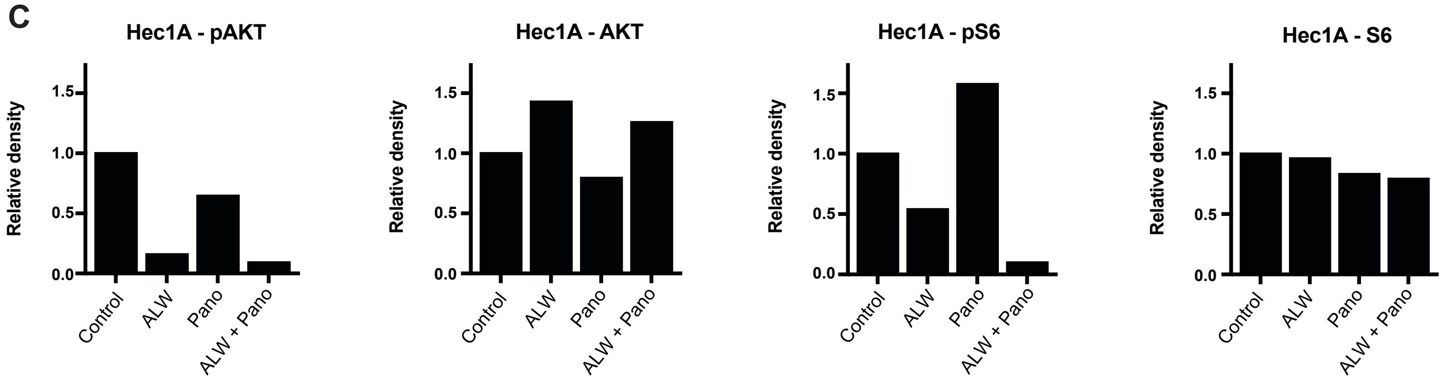

D

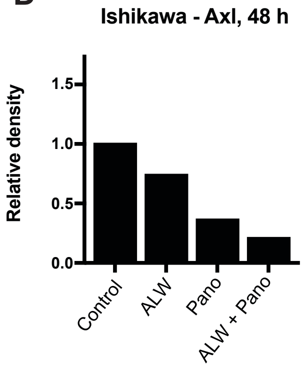

E

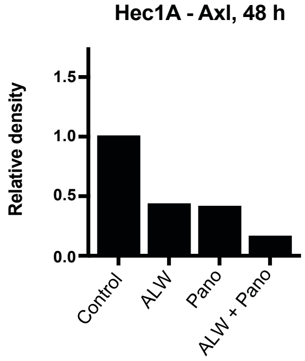

Relative density associated with Supplemental figures

S2C

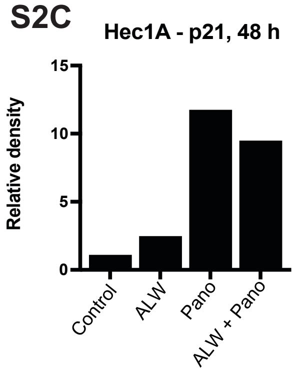

S3A

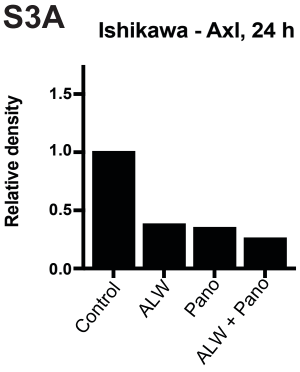

S3B

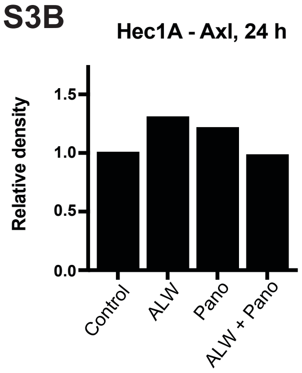

Supplement: Supplementary file 1 [file ijms-25-01278-s001.zip › ijms-2778903-figures.pdf]
